# Structure–function studies of the RNA polymerase II elongation complex

**DOI:** 10.1107/S0907444908039875

**Published:** 2009-01-20

**Authors:** Florian Brueckner, Karim-Jean Armache, Alan Cheung, Gerke E. Damsma, Hubert Kettenberger, Elisabeth Lehmann, Jasmin Sydow, Patrick Cramer

**Affiliations:** a Gene Center Munich and Center for Integrated Protein Science CIPSM, Department of Chemistry and Biochemistry, Ludwig-Maximilians-Universität München, 81377 Munich, Germany

**Keywords:** nucleic acids, transcription elongation, RNA polymerase II, nucleotide-addition cycle, translocation, transcription factor IIS, DNA-damage recognition, RNA-dependent RNA polymerase activity

## Abstract

RNA polymerase II (Pol II) is the eukaryotic enzyme that is responsible for transcribing all protein-coding genes into messenger RNA (mRNA). The mRNA-transcription cycle can be divided into three stages: initiation, elongation and termination. During elongation, Pol II moves along a DNA template and synthesizes a complementary RNA chain in a processive manner. X-ray structural analysis has proved to be a potent tool for elucidating the mechanism of Pol II elongation. Crystallographic snapshots of different functional states of the Pol II elongation complex (EC) have elucidated mechanistic details of nucleotide addition and Pol II translocation. Further structural studies in combination with *in vitro* transcription experiments led to a mechanistic understanding of various additional features of the EC, including its inhibition by the fungal toxin α-amanitin, the tunability of the active site by the elongation factor TFIIS, the recognition of DNA lesions and the use of RNA as a template.

## Abstract 2

X-ray crystallographic and complementary functional studies have contributed significantly to the current understanding of gene transcription. Here, recent structure–function studies on various aspects of the elongation phase of transcription are summarized.

## 1. Crystallography of the RNA polymerase II elongation complex

Crystallographic studies of Pol II from *Saccharomyces cerevisiae* were initiated in the Kornberg laboratory using the core enzyme, which consists of ten different protein subunits and has a total molecular weight of 469 kDa. Initial crystals (Fu *et al.*, 1999 ▶) could be dehydrated using a soaking procedure, which shrank the unit cell and improved the diffraction from 6 to 3 Å resolution (Cramer *et al.*, 2000 ▶). Phase information was obtained from multiple heavy-atom derivatives, including a six-Ta-atom cluster (Cramer *et al.*, 2000 ▶). Subsequently, a refined atomic model of the free core enzyme was obtained at 2.8 Å resolution (Cramer *et al.*, 2001 ▶) and as a tailed template elongation complex (EC) that revealed the DNA–RNA hybrid at 3.3 Å resolution (Gnatt *et al.*, 2001 ▶).

Crystals of the complete 12-subunit Pol II (molecular weight 514 kDa) including the two additional subunits Rpb4 and Rpb7 were subsequently obtained but displayed a high solvent content of 80% and only diffracted to around 4 Å resolution. This resulted in a backbone model of the complete enzyme (Armache *et al.*, 2003 ▶; Bushnell & Kornberg, 2003 ▶). In order to obtain an atomic model of the complete Pol II, atomic models of the core Pol II (at 2.8 Å resolution) and of the additional heterodimeric subcomplex Rpb4/7 (at 2.3 Å resolution) were combined and refined against the diffraction data obtained from a complete Pol II crystal at 3.8 Å resolution (Armache *et al.*, 2005 ▶). Further attempts were made to improve the diffraction of complete Pol II crystals, unfortunately without success. These included a search for a different crystal form, removal of the unstructured C-terminal tail of the largest Pol II subunit, cross-linking of the crystals with glutaraldehyde, controlled dehydration of the crystals, freezing in liquid ethane and crystal annealing. However, the resolution limit has recently been extended to 3.4 Å (Brueckner & Cramer, 2008 ▶) by optimizing the crystallization conditions and using the highly sensitive PILATUS 6M pixel detector, which has an increased signal-to-noise ratio (Broennimann *et al.*, 2006 ▶). The electron density was further improved by an improved processing and refinement strategy, which included the use of *XSCALE* with zero-dose extrapolation to compensate for radiation damage (Diederichs *et al.*, 2003 ▶) and *CNS* v.1.2 (Brunger, 2007 ▶) with a bulk-solvent parameter grid search for refinement and map calculation (Brueckner & Cramer, 2008 ▶). More recently, a data set extending to 3.0 Å resolution has been collected and the electron density was further enhanced by zonal scaling (Vassylyev, Vassylyeva, Perederina *et al.*, 2007 ▶, and unpublished results).

**Figure 1:**
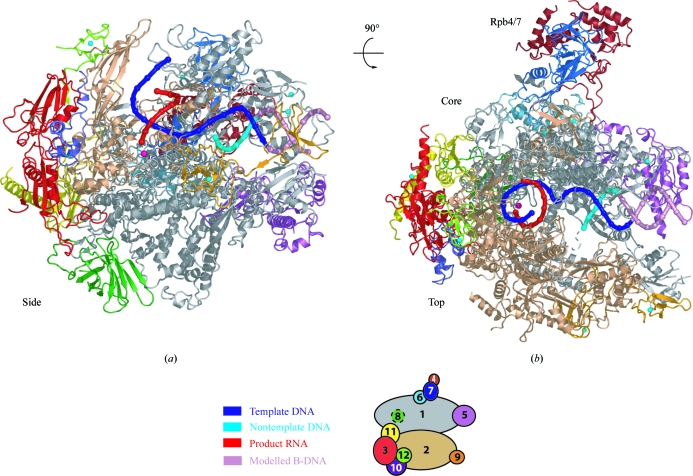
Structural overview of the complete 12-subunit RNA polymerase II elongation complex (Kettenberger *et al.*, 2004 ▶). Two views are shown of a ribbon model of the protein subunits and nucleic acids, a side view (*a*) and a top view (*b*), related by a 90° rotation around a horizontal axis. The polymerase subunits Rpb1–Rpb12 are coloured according to the key shown below. Template DNA, nontemplate DNA and product RNA are shown in blue, cyan and red, respectively. P atoms are indicated as spheres and extrapolated B-form downstream DNA is coloured light pink. Eight zinc ions and the active-site magnesium ion are depicted as cyan spheres and a magenta sphere, respectively. This colour code is used throughout. Secondary-structure assignments for Pol II are according to Cramer *et al.* (2001 ▶) and Armache *et al.* (2005 ▶). This figure was adapted from Kettenberger *et al.* (2004 ▶) with modifications.

Structural studies of the Pol II EC have elucidated the mechanism of RNA elongation. Electron microscopy first revealed the point of DNA entry into the Pol II cleft (Poglitsch *et al.*, 1999 ▶). The first reported crystal structure of a Pol II–nucleic acid complex was that of the core Pol II transcribing a DNA template with a single-stranded ‘tail’ at one 3′ end (tailed template; Gnatt *et al.*, 2001 ▶). This structure revealed an 8–9 base-pair DNA–RNA hybrid in the active centre. Comparison with the high-resolution core Pol II structure (Cramer *et al.*, 2000 ▶, 2001 ▶) revealed protein-surface elements predicted to play functional roles. Subsequently, polymerase EC structures were obtained using different kinds of synthetic DNA–RNA scaffolds. The complete Pol II EC structure contained a mismatch bubble scaffold with upstream and downstream DNA duplexes and RNA annealed to a central mismatched bubble region (Figs. 1 ▶ and 2 ▶; Kettenberger *et al.*, 2004 ▶). The atomic model of the complete 12-subunit Pol II (Armache *et al.*, 2005 ▶) was crucial in obtaining high-quality difference electron-density maps for Pol II ECs after molecular replacement (Armache *et al.*, 2005 ▶; Kettenberger *et al.*, 2004 ▶). However, the upstream DNA duplex and the non­template strand in the bubble region were disordered in the crystal structure. Reduced scaffolds lacking these disordered parts (‘minimal nucleic acid scaffolds’) were used to determine structures of the core Pol II EC (Westover *et al.*, 2004*a* ▶,*b* ▶). The synthetic scaffold EC structures revealed the exact location of the downstream DNA and several nucleotides upstream of the hybrid (Figs. 1 ▶ and 2 ▶). Mechanisms were suggested for how Pol II unwinds downstream DNA and how it separates the RNA product from the DNA template at the end of the hybrid. In both cases, Pol II-induced distortion of the nucleic acid duplexes and steric hindrance by Pol II surface loops seem to play important roles.

## 2. Nucleotide incorporation

The events required for the addition of a nucleotide to the product RNA form a cyclic process referred to as the ‘nucleotide-addition cycle’ (NAC; Fig. 3 ▶). RNA extension begins with the binding of a nucleoside triphosphate (NTP) substrate to the EC that is formed by the polymerase, DNA and RNA. Catalytic addition of the nucleotide to the growing RNA 3′ end then releases a pyrophosphate ion. Finally, translocation of DNA and RNA frees the substrate site for binding of the next NTP.

**Figure 2:**
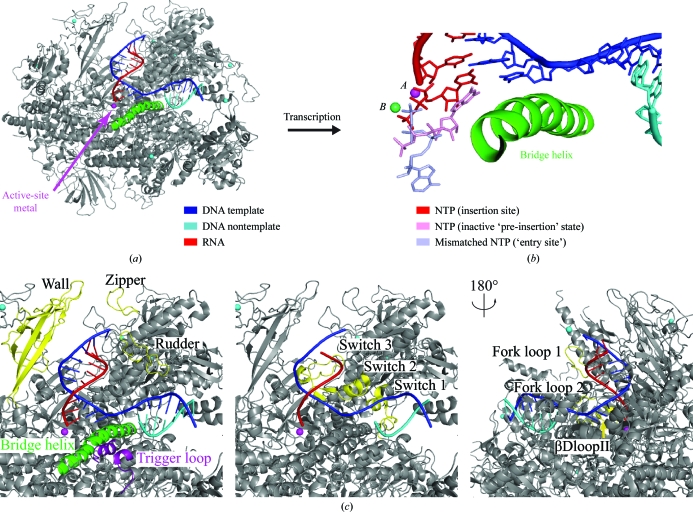
Structural details of the Pol II elongation complex. (*a*) Overview of the EC structure (Kettenberger *et al.*, 2004 ▶). The view is as in Fig. 1 ▶(*a*). (*b*) Superposition of NTP-binding sites [red, insertion site (Westover *et al.*, 2004*a* ▶; Wang *et al.*, 2006 ▶); violet, entry site (Westover *et al.*, 2004*a* ▶); pink, inactive pre-insertion-like state in which the triphosphate is too far from the catalytic metal ion *A* to allow incorporation (Kettenberger *et al.*, 2004 ▶)]. (*c*) Functional Pol II surface elements in the EC highlighted in yellow. This figure was adapted from Cramer *et al.* (2008 ▶).

**Figure 3:**
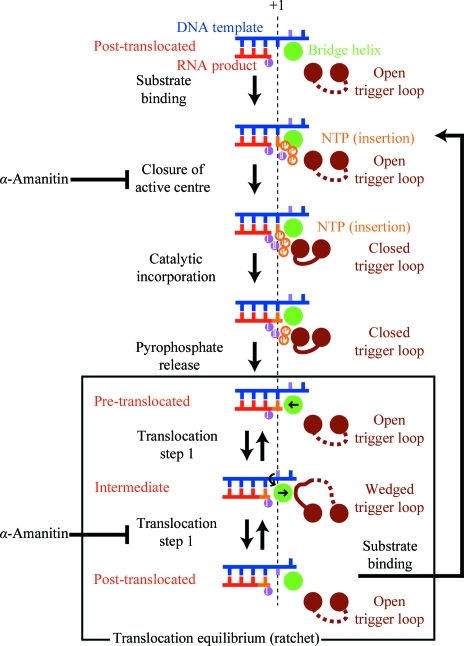
Schematic representation of the extended model for the nucleotide-addition cycle (NAC). The vertical dashed line indicates register +1. The steps where α-amanitin interferes with the NAC are indicated. For details, refer to the text. This figure was adapted from Brueckner & Cramer (2008 ▶) with modifications.

The NAC was studied with additional structures of Pol II ECs that included the NTP substrate (Fig. 2 ▶ *b*; Westover *et al.*, 2004*a* ▶; Wang *et al.*, 2006 ▶; Kettenberger *et al.*, 2004 ▶). The NTP was crystallographically trapped in the insertion site (Wang *et al.*, 2006 ▶; Westover *et al.*, 2004*a* ▶), which is apparently occupied during catalysis, and also in an overlapping slightly different location, suggesting an inactive NTP-bound pre-insertion state of the enzyme (Fig. 3 ▶; Kettenberger *et al.*, 2004 ▶). The NTPs in both states form Watson–Crick interactions with a base in the DNA-template strand. Binding of the NTP to the insertion site involves folding of the trigger loop (Fig. 2 ▶ *c*; Wang *et al.*, 2006 ▶), a mobile part of the active centre that was first observed in free bacterial RNA polymerase (Vassylyev *et al.*, 2002 ▶) and in the Pol II–TFIIS complex (Kettenberger *et al.*, 2003 ▶). Folding of the trigger loop closes the active site and may be involved in selection of the correct NTP (Fig. 3 ▶). The NTP-complex structures revealed contacts of the nucleotide with the polymerase, which explain the discrimination of ribonucleotides against deoxyribonucleotides, and provided insights into the selection of the nucleotide complementary to the templating DNA base.

Catalytic nucleotide incorporation apparently follows the two-metal-ion mechanism suggested for all polymerases (Steitz, 1998 ▶). The Pol II active site contains a persistently bound metal ion (metal *A*) and a second mobile metal ion (metal *B*) (Cramer *et al.*, 2001 ▶). Metal *A* is held in place by three invariant aspartate side chains and binds the RNA 3′ end (Cramer *et al.*, 2001 ▶), whereas metal *B* binds the NTP triphosphate moiety (Westover *et al.*, 2004*a* ▶).

Recent studies of functional complexes of the bacterial RNA polymerase revealed the close conservation of the EC structure (Vassylyev, Vassylyeva, Perederina *et al.*, 2007 ▶) and provided additional insights into nucleotide incorporation (Vassylyev, Vassylyeva, Zhang *et al.*, 2007 ▶). As for Pol II, NTP binding to the insertion site can induce folding of the trigger loop. However, in the presence of the antibiotic streptolydigin the NTP binds in the inactive pre-insertion state, in which the triphosphate and metal *B* are too far from metal *A* to permit catalysis. This finding supported a two-step mechanism of nucleotide incorporation (Fig. 3 ▶; Vassylyev, Vassylyeva, Zhang *et al.*, 2007 ▶; Kettenberger *et al.*, 2004 ▶). The NTP first binds in the inactive state to an open active-centre conformation. Complete folding of the trigger loop then leads to closure of the active centre, delivery of the NTP to the insertion site and catalysis. An alternative model for nucleotide addition involves binding of the NTP to a putative entry site in the pore, in which the nucleotide base is oriented away from the DNA template, and possible rotation of the NTP around metal ion *B* directly into the insertion site (Westover *et al.*, 2004*a* ▶).

**Figure 4:**
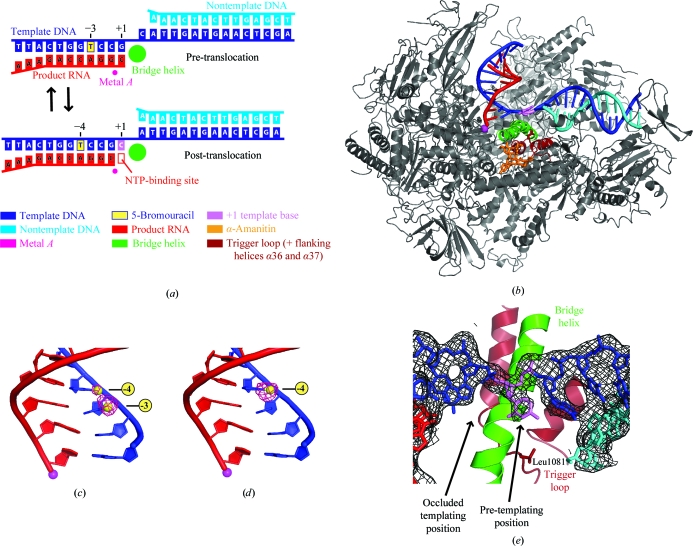
Structure of the α-amanitin-inhibited Pol II elongation complex. (*a*) Pre-translocation and post-translocation states of the EC. The nucleic acid scaffold used is depicted schematically with respect to the active-site metal ion *A* (magenta). The colour key is used throughout. (*b*) Overview of the α-amanitin-inhibited Pol II EC structure. The view is as in Fig. 1 ▶(*a*). α-Amanitin (stick model), nucleic acids (base in pre-templating position as a stick model), metal *A*, the bridge helix and the trigger loop (Leu1081 as a stick model) are highlighted using the colour key in (*a*). Part of the protein is omitted for clarity. (*c*, *d*) Bromine anomalous difference Fourier maps (pink net) of the free EC (*c*) and the α-amanitin-inhibited EC (*d*). Br atoms are depicted as yellow spheres and their positions are indicated. The view is rotated by 90° around a vertical axis compared with (*b*). (*e*) The +1 DNA-template base adopts a pre-templating position. The initial unbiased *F*_o_ − *F*_c_ difference map for the nucleic acids is shown around the +1 position and is contoured at 2.5σ. The +1 base in the pre-templating site is highlighted in violet. The view is rotated by 90° around a horizontal axis compared with (*b*). This figure was adapted from Brueckner & Cramer (2008 ▶).

After nucleotide incorporation, the substrate-binding site is occupied by the 3′ end of the product RNA and the EC adopts the pretranslocation state. Pol II translocates by a one-base-pair step in order to free the substrate-binding site for the next round of incorporation and thereby reaches the post-trans­location state (Fig. 4 ▶ *a*). The Brownian ratchet model of translocation assumes that Brownian motion gives rise to oscillation of the EC between pre-translocation and post-translocation states, establishing the translocation equilibrium. Substrate NTPs can only bind in the post-translocation state and would act like the pawl of a ratchet. X-ray structural evidence for the existence of the translocation equilibrium was recently obtained with an EC labelled with 5-bromouracil in the template strand (Figs. 4 ▶ *a* and 4 ▶ *c*; Svetlov & Nudler, 2008 ▶; Brueckner & Cramer, 2008 ▶). Soaking the crystals with the preserved translocation equilibrium with the inhibitor α-­amanitin resulted in the structure of the α-amanitin-inhibited EC at 3.4 Å resolution, which was suggested to represent a translocation intermediate (Fig. 4 ▶ *b*). In this putative intermediate the DNA–RNA hybrid adopts a post-translocation state (Fig. 4 ▶ *d*), whereas the state of the downstream DNA is intermediary between pre-translocation and post-translocation. The template base entering the active centre (the templating base) was found in a new ‘pre-templating’ position above the central bridge helix (Fig. 4 ▶ *e*). Two Pol II elements, the trigger loop and the bridge helix, were observed in new conformations, suggesting their involve­ment in facilitating translocation. α-Amanitin apparently traps the trigger loop and bridge helix in these conformations with direct and indirect contacts, thereby inhibiting nucleotide incorporation and translocation. An independent study also revealed the direct contact between the trigger loop and α-­amanitin and additionally showed a role of the trigger loop in substrate selection and fidelity (Kaplan *et al.*, 2008 ▶).

## 3. Obstacles during elongation

**Figure 5:**
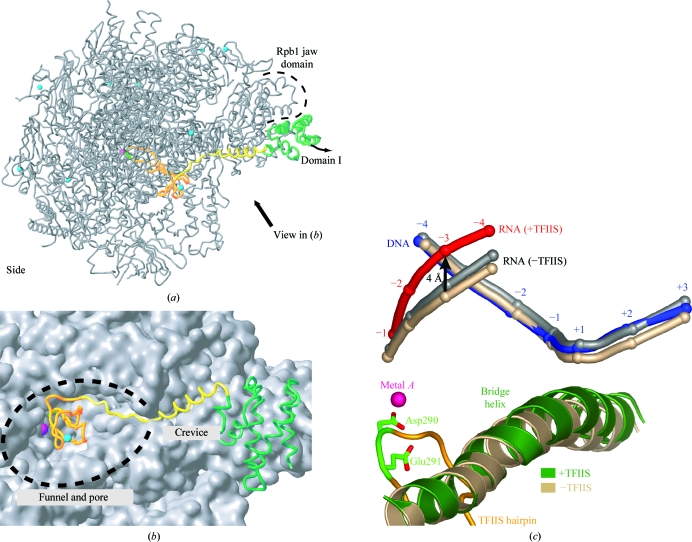
Structures of Pol II (*a*, *b*) and the Pol II EC (*c*) in complex with TFIIS. (*a*) Ribbon diagram of the Pol II–TFIIS complex backbone model (Kettenberger *et al.*, 2003 ▶). The 12 subunits of Pol II are shown in silver. A pink sphere marks the location of the active-site metal ion *A*. Eight structural zinc ions in Pol II and one zinc ion in TFIIS are depicted as cyan spheres. The view is as in Fig. 1 ▶(*a*). (*b*) Binding of TFIIS to the jaw, crevice, funnel and pore. TFIIS is shown as a ribbon model on the molecular surface of Pol II. The view is from the bottom face, as indicated in (*a*). (*c*) TFIIS-induced RNA realignment (Kettenberger *et al.*, 2004 ▶). Selected elements in the Pol II active centre that move upon TFIIS binding are shown. The bridge helix, DNA and RNA in the Pol II–bubble-RNA–TFIIS complex are shown in green, blue and red, respectively. The TFIIS hairpin is in orange, with the two acidic functionally essential and invariant residues in green. Nucleic acids in the Pol II–bubble-RNA complex structure after superposition of residues in the active-site aspartate loop or in switch 2 are shown in beige and grey, respectively. Switch 2 moves slightly upon TFIIS binding (Kettenberger *et al.*, 2003 ▶), explaining the difference in the two superpositions. This figure was adapted from Kettenberger *et al.* (2003 ▶, 2004 ▶).

During active transcription, Pol II must overcome intrinsic DNA-arrest sites, which are generally rich in A·T base pairs and pose a natural obstacle to transcription. At such sites, Pol II moves backwards along DNA and RNA, resulting in extrusion of the RNA 3′ end through the polymerase pore beneath the active site and transcriptional arrest. The RNA-cleavage stimulatory factor TFIIS can rescue an arrested polymerase by creating a new RNA 3′ end at the active site from which transcription can resume. The mechanism of TFIIS function was elucidated from the structures of Pol II and a Pol II EC in complex with TFIIS (Fig. 5 ▶; Kettenberger *et al.*, 2003 ▶, 2004 ▶). TFIIS inserts a hairpin into the polymerase pore and complements the active site with acidic residues, changes the enzyme conformation and repositions the RNA transcript (Kettenberger *et al.*, 2003 ▶, 2004 ▶). These studies supported the idea that the Pol II active site is tunable, as it can catalyze different reactions, including RNA synthesis and RNA cleavage (Kettenberger *et al.*, 2003 ▶; Sosunov *et al.*, 2003 ▶).

**Figure 6:**
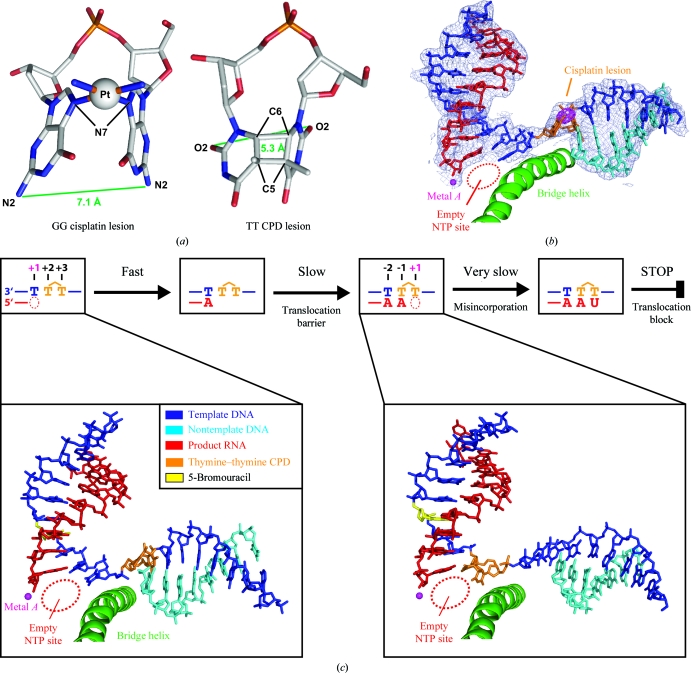
Mechanisms of DNA-damage recognition. (*a*) Structures of two different DNA dinucleotide lesions. The maximum lateral dimensions are indicated in green. (*b*) Structure of a cisplatin-damaged Pol II elongation complex (Damsma *et al.*, 2007 ▶). Final 2*F*_o_ − *F*_c_ electron-density map for the nucleic acids is shown (blue, contoured at 1.0σ). Anomalous difference Fourier map reveals the location of the Pt atom (magenta, contoured at 15σ). The cisplatin lesion is located outside of the active centre at positions +2/+3. This panel was adapted from Damsma *et al.* (2007 ▶). (*c*) Simplified mechanism of CPD DNA-damage recognition by Pol II. At the top, a schematic is shown that depicts the last few steps before Pol II stalling. At the bottom, nucleic acid structures in Pol II ECs containing a thymine–thymine CPD lesion before (left) and in the active site (right) are shown. DNA template, DNA nontemplate and RNA strands are shown in blue, cyan and red, respectively. The CPD is shown as a stick model in orange. The active-site magnesium ion (metal *A*) is depicted as a magenta sphere. This panel was adapted from Brueckner & Cramer (2007 ▶).

Other obstacles to transcription are bulky lesions in the DNA-template strand, *e.g.* the UV-light-induced cyclobutane pyrimidine dimer (CPD), or intrastrand cross-links induced by the anticancer drug cisplatin (Fig. 6 ▶). Bulky DNA lesions can block transcription and replication and lead to mutations that can cause cancer (Mitchell *et al.*, 2003 ▶). Cells can eliminate bulky DNA lesions slowly by genome-wide nucleotide-excision repair (NER). However, for rapid and efficient repair cells use an NER subpathway referred to as transcription-coupled DNA repair (TCR). TCR specifically removes lesions such as CPDs from the DNA strand transcribed by Pol II (Saxowsky & Doetsch, 2006 ▶). It is thought that only those lesions that can stably stall Pol II trigger TCR. CPDs are bulky lesions that lead to Pol II stalling, but other types of damage, such as oxidative damage, can be bypassed by Pol II and would escape TCR (Charlet-Berguerand *et al.*, 2006 ▶). Pol II stalling apparently triggers TCR by the recruitment of a transcription-repair coupling factor (Rad26/CSB in yeast/human) and factors required for subsequent steps of nucleotide-excision repair, including TFIIH, which unwinds DNA, and endo­nucleases, which incise the DNA strand on either side of the lesion (Saxowsky & Doetsch, 2006 ▶; Svejstrup, 2002 ▶; Selby *et al.*, 1997 ▶; Tremeau-Bravard *et al.*, 2004 ▶; Mu & Sancar, 1997 ▶). The DNA gap obtained is subsequently filled by DNA synthesis and ligation (Sancar, 1996 ▶; Prakash & Prakash, 2000 ▶).

## 4. Mechanisms of DNA-damage recognition

To study the mechanism of DNA-damage recognition by Pol II, expertise in the synthesis of lesion-containing DNA (the group of T. Carell at the University of Munich) was combined with expertise in preparing functional crystallization-grade ECs of the complete 12-subunit Pol II (our group). Bulky DNA lesions were introduced into the DNA-template strand at several different positions around the polymerase active site and the resulting Pol II ECs were studied structurally (Fig. 6 ▶) and in RNA-elongation assays (Brueckner & Cramer, 2007 ▶; Brueckner *et al.*, 2007 ▶). The highly reproducible and clean system for reconstituting defined fully functional Pol II ECs is very useful for a detailed structure–function analysis of many more aspects of the transcription mechanism in the future.

Pol II stalls when a CPD in the DNA-template strand reaches the enzyme active site after nucleotide incorporation opposite both CPD thymines (Fig. 6 ▶ *c*; Tornaletti *et al.*, 1997 ▶; Mei Kwei *et al.*, 2004 ▶). However, it is not obvious how the CPD can reach the active site since transfer of a DNA-template base from the downstream position +2 to the nucleotide-insertion site at +1 over the polymerase bridge helix normally requires twisting of the base by 90° and such twisting is not possible for the CPD thymines, since they are covalently linked. In addition, accommodation of the 3′-­thymine in the templating position would lead to a severe clash of the 5′-thymine with the bridge helix which lines the front end of the active centre. Therefore, instead of using the 3′-thymine of the CPD as a template, Pol II apparently incorporates AMP in a nontemplated manner opposite the 3′-­thymine of the CPD, according to an A-rule known for DNA polymerases, while the CPD is suspended outside of the active centre (Brueckner *et al.*, 2007 ▶; Damsma *et al.*, 2007 ▶; Taylor, 2002 ▶; Li *et al.*, 2004 ▶).

After the nontemplated AMP addition opposite the 3′-­thymine, the CPD can enter the active site and is stably accommodated at positions −1/+1 of the template strand, forming a Watson–Crick base pair between the 3′-thymine and the adenine at the 3′ end of the product RNA (Fig. 6 ▶ *c*). Now only UMP can be incorporated opposite the 5′-thymine (Brueckner *et al.*, 2007 ▶; Mei Kwei *et al.*, 2004 ▶). The UMP misincorporation is very slow and is the rate-limiting step in reaching the stalled state (Brueckner *et al.*, 2007 ▶). Specific UMP misincorporation may arise from the unusual position of the CPD 5′-thymine, which adopts a wobble position with respect to the base in the undamaged complex (Brueckner *et al.*, 2007 ▶). The wobbled 5′-thymine can form two hydrogen bonds to UTP, but not to other NTPs. Pol II stalls because translocation of the CPD 5′-thymine–uracil mismatch base pair from position +1 to position −1 is strongly disfavoured. This translocation event would move the damage-containing mismatch into the −1 position of the DNA–RNA hybrid, resulting in a distortion that is likely to destabilize the EC (Kireeva *et al.*, 2000 ▶). Replacement of the misincorporated UMP by AMP in an artificial scaffold enables CPD bypass (Brueckner *et al.*, 2007 ▶). Thus, Pol II stalling requires CPD-directed misincorporation and distortions arising from the CPD alone are insufficient to cause Pol II stalling. Indeed, a T·U mismatch base pair alone was sufficient to stall the vast majority of Pol II complexes (Brueckner *et al.*, 2007 ▶). In contrast, DNA polymerases can correctly incorporate adenine opposite both CPD thymines and, depending on the type of polymerase, this can lead to stalling or lesion bypass (Li *et al.*, 2004 ▶; Ling *et al.*, 2003 ▶).

The anticancer drug cisplatin [*cis*-diamminedichloro­platinum(II)] forms 1,2-d(GpG) DNA intrastrand cross-links (cisplatin lesions) that stall Pol II and trigger transcription-coupled DNA repair (Wang & Lippard, 2005 ▶; Kartalou & Essigmann, 2001 ▶; Corda *et al.*, 1991 ▶, 1993 ▶; Tornaletti *et al.*, 2003 ▶; Jung & Lippard, 2006 ▶). Whereas in the CPD lesion two neighbouring thymine bases are covalently linked with a cyclobutane ring including the C5 and C6 atoms, in a cisplatin lesion the Pt atom coordinates the N7 atoms of two adjacent guanines in a DNA strand (Fig. 6 ▶ *a*). The cisplatin lesion can be stably accommodated in a Pol II EC at position +2/+3 of the template strand, but translocation to position +1/+2 is dis­favoured (Fig. 6 ▶ *b*; Damsma *et al.*, 2007 ▶); these are both also the case for the CPD lesion. There is strong evidence that adenine is incorporated in a nontemplated fashion opposite the cisplatin 3′-guanine, as proposed for the CPD lesion. However, unlike the CPD lesion, the cisplatin lesion cannot be stably accommodated in the active site (positions −1/+1). There are two possible causes. Firstly, the cisplatin lesion is a more bulky dinucleotide lesion than the CPD lesion. The maximum lateral dimension is 7.2 Å (N2–N2 distance), compared with 5.3 Å (O2–O2 distance) for the CPD lesion (Fig. 6 ▶ *a*). Modelling suggested that a conformational change of the bridge helix would be required to accommodate the lesion in the active site. Secondly, a G·A mismatch base pair would be formed at position −1 in contrast to a stabilizing T·A base pair in the case of the CPD lesion.

In conclusion, the mechanism of recognition by transcribing Pol II is different for the two dinucleotide lesions. At a cisplatin lesion, Pol II stalls because the lesion cannot be delivered to the active site, whereas it stalls at a CPD lesion after delivery to the active site and specific UMP misincorporation opposite the 5′-thymine. Bypass of the CPD lesion is only possible by artificially replacing the resulting T·U mismatch by a T·A match. Remarkably, bypass of the cisplatin lesion is also possible, but only by artificially providing a starting transcript that extends at least up to the 3′-guanine. In this case, bypass is even possible in presence of a G·A mismatch with the 3′-­guanine of the cisplatin dimer.

## 5. RNA as a template for Pol II

Although Pol II generally uses DNA as a template, there is also evidence that Pol II can use RNA templates. Recent structures have shown that an RNA template–product duplex can bind to the site normally occupied by the DNA–RNA hybrid and provided the structural basis for the phenomenon of RNA-dependent RNA synthesis by Pol II (Lehmann *et al.*, 2007 ▶). Complementary *in vitro* enzyme assays revealed that the RNA-dependent RNA polymerase (RdRP) activity resides in the site used during transcription, but is slower and less processive than the DNA-dependent activity. The RdRP activity of Pol II provides a missing link in molecular evolution, because it suggests that Pol II evolved from an ancient replicase that duplicated RNA genomes. There is compelling evidence that the ancient RdRP activity of Pol II is still relevant for the replication of the RNA genome of the hepatitis δ virus (HDV) and it may also be used in certain cellular processes as many organisms lack dedicated single-subunit RdRPs.

## 6. Conclusion

Combining X-ray crystallographic analysis of Pol II ECs with *in vitro* transcription experiments allowed exploration of the basic mechanisms of transcription elongation, including the nucleotide-addition cycle, and additional features such as the mechanism of TFIIS function, DNA-damage recognition and RNA-templated RNA synthesis. Further aspects of transcription elongation still await further characterization using the available system. Further investigations could focus on the regulation by additional protein factors or RNA molecules, transcriptional mutagenesis and fidelity and the effect of other kinds of DNA lesions, *e.g.* oxidative lesions, to name a few.
